# Microparticles Carrying Sonic Hedgehog Are Increased in Humans with Peripheral Artery Disease

**DOI:** 10.3390/ijms19123954

**Published:** 2018-12-09

**Authors:** Igor Giarretta, Ilaria Gatto, Margherita Marcantoni, Giulia Lupi, Diego Tonello, Eleonora Gaetani, Dario Pitocco, Roberto Iezzi, Addolorata Truma, Angelo Porfidia, Adriana Visonà, Paolo Tondi, Roberto Pola

**Affiliations:** 1 Department of Medicine, Fondazione Policlinico Universitario A. Gemelli IRCCS, Università Cattolica del Sacro Cuore, 00168 Rome, Italy; igor.giarretta@unicatt.it (I.G.); ilariagatto@yahoo.it (I.G.); margheritamarcantoni@yahoo.it (M.M.); giu.lu3@gmail.com (G.L.); eleonora.gaetani@unicatt.it (E.G.); ada.truma@gmail.com (A.T.); porfidia.a@gmail.com (A.P.); paolotondi@yahoo.it (P.T.); 2 Unit of Angiology, S. Giacomo Hospital, 31033 Castelfranco Veneto, Italy; diegotonello@yahoo.com (D.T.); adrianavisona@gmail.com (A.V.); 3 Diabetology Unit, Fondazione Policlinico Universitario A. Gemelli IRCCS, Università Cattolica del Sacro Cuore, 00168 Rome, Italy; dario.pitocco@unicatt.it; 4 Department of Radiological Sciences, Fondazione Policlinico Universitario A. Gemelli IRCCS, Università Cattolica del Sacro Cuore, 00168 Rome, Italy; roberto.iezzi.md@gmail.com

**Keywords:** sonic hedgehog, microparticles, peripheral artery disease

## Abstract

Sonic hedgehog (Shh) is a prototypical angiogenic agent with a crucial role in the regulation of angiogenesis. Experimental studies have shown that Shh is upregulated in response to ischemia. Also, Shh may be found on the surface of circulating microparticles (MPs) and MPs bearing Shh (Shh + MPs) have shown the ability to contribute to reparative neovascularization after ischemic injury in mice. The goal of this study was to test the hypothesis that, in humans with peripheral artery disease (PAD), there is increased number of circulating Shh + MPs. This was done by assessing the number of Shh + MPs in plasma of patients with PAD and control subjects without PAD. We found significantly higher number of Shh + MPs in plasma of subjects with PAD, compared to controls, while the global number of MPs—produced either by endothelial cells, platelets, leukocytes, and erythrocytes—was not different between PAD patients and controls. We also found a significant association between the number of Shh + MPs and the number of collateral vessels in the ischemic limbs of PAD patients. Interestingly, the concentration of Shh protein unbound to MPs—which was measured in MP-depleted plasma—was not different between subjects with PAD and the controls, indicating that, in the setting of PAD, the call for Shh recapitulation does not lead to secretion of protein into the blood but to binding of the protein to the membrane of MPs. These findings provide novel information on Shh signaling during ischemia in humans, with potentially important biological and clinical implications.

## 1. Introduction

Sonic hedgehog (Shh) is a morphogen belonging to the hedgehog (Hh) family of proteins and is crucial during embryonic development [1]. In post-natal life, the reactivation of the Shh pathway has been observed in various organs and tissues after injury and during regeneration, as well as in tumors [2]. In multiple experimental models, it has been demonstrated that ischemia induces the recapitulation of the Shh pathway, triggering a variety of responses in endothelial cells (ECs), endothelial-progenitor cells (EPCs), smooth muscle cells (SMCs), and fibroblasts, and promoting angiogenesis and vasculogenesis [3,4,5,6,7]. 

Microparticles (MPs) are small plasma membrane fragments shed by cells after blebbing due to cell activation and/or apoptosis. They play an important role in cell to cell communication because of their ability to act at distant site as well as locally, and to propagate the functional antigens of their parent cell [8]. Angiogenesis is among the processes that may be regulated by MPs [9].

There is experimental evidence that MPs harboring Shh (Shh + MPs) may modulate the nitric oxide (NO) pathway, regulate the production of pro-angiogenic factors and the up-regulation of proteins involved in cell adhesion, and orchestrate several processes related to cell proliferation, differentiation and angiogenesis [10]. However, such findings have been generated only in pre-clinical settings. 

The experiments presented in this paper have tested the hypothesis that, in humans with peripheral artery disease (PAD)—a prototypical ischemic cardiovascular pathology [11]—there is increased production of Shh, similar to what has been previously demonstrated by our groups in various animal models of ischemia [3,12,13,14]. Our findings demonstrate increased levels of Shh bound to circulating MPs in the plasma of subjects with PAD, compared to controls, while Shh concentration in MP-depleted plasma is similar in subjects with and without PAD.

## 2. Results

The main demographic and clinical characteristics of the studied population are summarized in Table 1. Briefly, PAD patients and controls did not differ in terms of age (71.4 ± 9.4 vs. 70.3 ± 8.2, *p* = n.s.) and presence of diabetes (44.0% vs. 34.0%, *p* = n.s.), dyslipidemia (72.0% vs. 60.0%, *p* = n.s.), and hypertension (80.0% vs. 80.0%, *p* = n.s.). PAD patients were more frequently men (74.0% vs. 54.0%, *p* < 0.05) and smokers (34.0% vs. 14.0%, *p* < 0.05). History of myocardial infarction (MI) and stroke was reported more frequently by PAD patients than controls (42.0% vs. 8.0%, *p* < 0.01 and 12.0% vs. 0.0%, *p* < 0.05, respectively). Regarding pharmacological therapy, 84.0% of PAD patients were on single anti-platelet treatment (SAPT) (either aspirin, clopidogrel, cilostazol, or ticlopidine) and 10.0% were on dual antiplatelet therapy (DAPT). On the other hand, 48.0% of controls were on SAPT and none was in DAPT. The use of statins, beta-blockers, and ACE inhibitors/ARB was not statistically different between patients and controls (72.0% vs. 56.0%, 54.0% vs. 52.0%, and 46.0% vs. 38.0%, respectively, *p* = n.s.). Regarding disease severity, 15 PAD patients were in Leriche–Fontaine stage IIa (30.0%), 15 were in stage IIb (30.0%), 10 in stage III (20.0%), and 10 in stage IV (20.0%).

**Figure 1:**
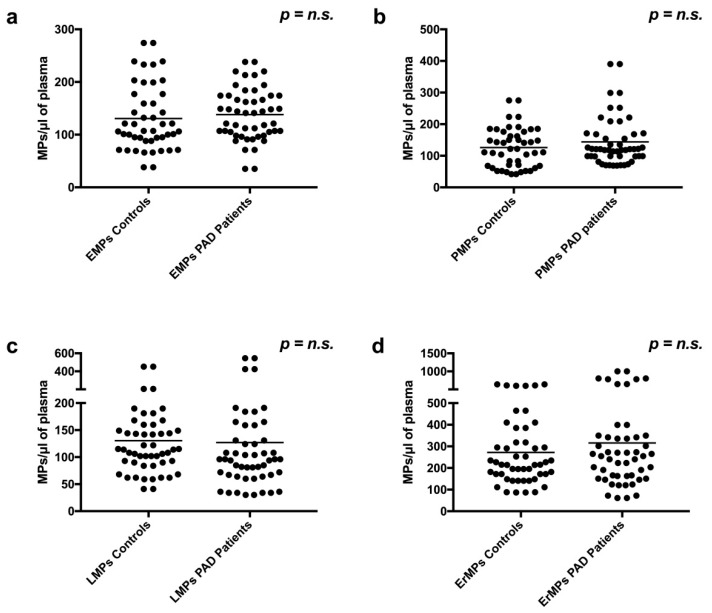
Global number of endothelial microparticles (EMPs) (**a**), platelet-derived microparticles (PMPs) (**b**), leucocyte-derived microparticles (LMPs) (**c**), and erythrocyte-derived microparticles (ErMPs) (**d**) in plasma of peripheral artery disease (PAD) patients and controls. *p*: *p*-value; *n.s*.: not significant. Black horizontal lines in panels indicate median values.

When looking at the global number of MPs of endothelial origin (EMPs), no significant difference was found between controls and PAD patients (130.6 ± 62.2 vs. 138.2 ± 49.2, *p* = n.s.) (Figure 1a). Similarly, no differences were found in terms of global number of platelet-derived MPs (PMPs) (126.1 ± 61.6 vs. 143.9 ± 77.1, *p* = n.s.) (Figure 1b), leucocyte-derived MPs (LMPs) (130.5 ± 79.0 vs. 126.9 ± 116.6, *p* = n.s.) (Figure 1c), and erythrocyte-derived MPs (ErMPs) (271.6 ± 159.5 vs. 315.5 ± 238.7, *p* = n.s.) (Figure 1d).

**Figure 2:**
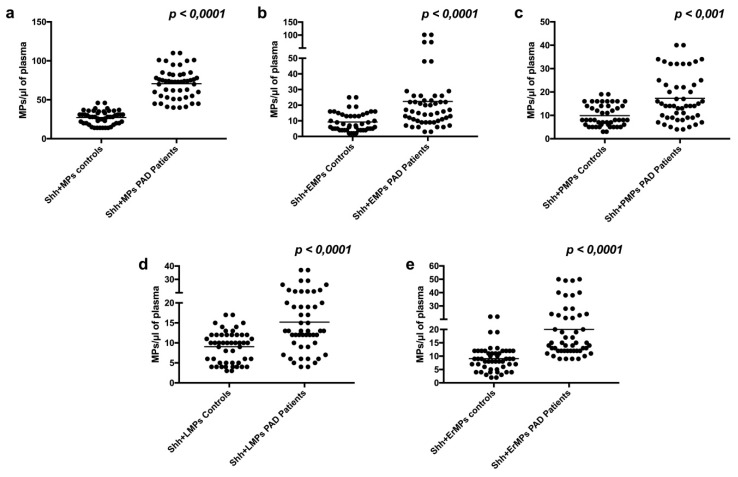
Global number of Sonic hedgehog (Shh) + microparticles (MPs) in PAD patients and controls (**a**); number of Shh + EMPs (**b**), Shh + PMPs (**c**), Shh + LMPs (**d**), and Shh + ErMPs (**e**) in PAD patients and controls. *p*: *p*-value; *n.s*.: not significant. Black horizontal lines in panels indicate median values.

In contrast, we found a significantly higher number of Shh + MPs in the plasma of subjects with PAD, compared to controls (70.5 ± 19.0 vs. 27.2 ± 8.5, *p* < 0.001) (Figure 2a). This increment was due to an increased number of Shh + EMPs (22.3 ± 21.8 vs. 9.2 ± 6.0, *p* < 0.001) (Figure 2b), as well as to an increased number of Shh + MPs derived from platelets (17.3 ± 10.1 vs. 9.9 ± 4.6, *p* < 0.001) (Figure 2c), leukocytes (15.2 ± 7.9 vs. 9.0 ± 3.9, *p* < 0.001) (Figure 2d), and erythrocytes (20.0 ± 11.8 vs. 9.8 ± 4.6, *p* < 0.001) (Figure 2e). 

A summary of the results of these studies is presented in Table 2.

**Figure 3:**
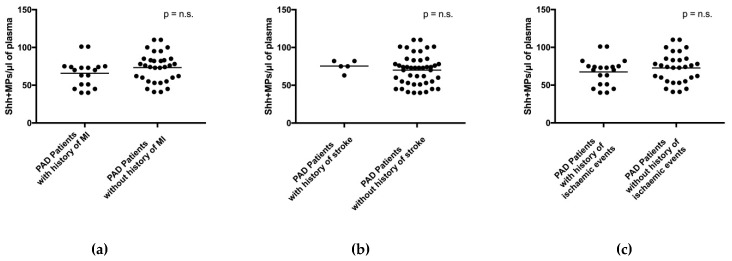
Global number of Shh + MPs in PAD patients with and without history of myocardial infarction (MI) (**a**), with and without history of stroke (**b**), and with and without history of either MI and/or stroke (**c**). *p*: *p*-value; *n.s*.: not significant. Black horizontal lines in panels indicate median values.

Since in subjects with PAD the number of circulating Shh + MPs may be affected by the presence of other diseases—in particular coronary artery disease (CAD) and cerebrovascular disease—we separately analyzed PAD patients with and without history of MI, with and without history of ischemic stroke, and with and without history of either MI and/or stroke. We did not find significant differences between groups (Figure 3).

**Figure 4:**
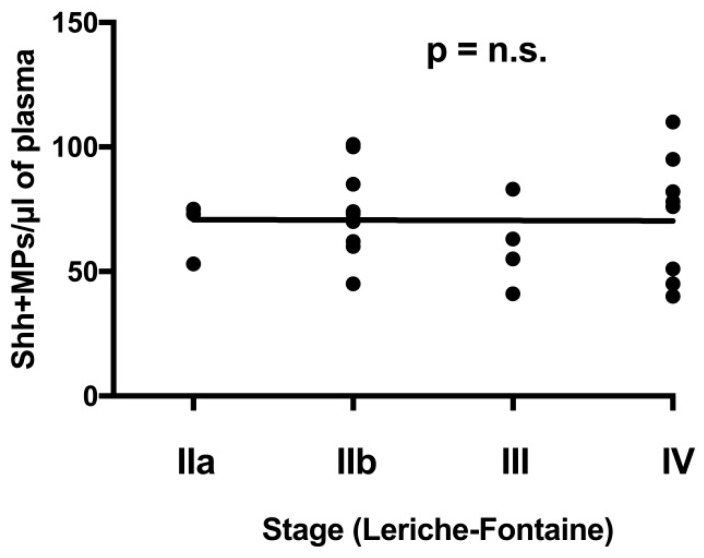
Global number of Shh + MPs in PAD patients according to disease stage. Black horizontal line indicates the interpolate curve.

We also looked at the number of Shh + MPs in PAD subjects at various stages of the disease, according to the Leriche–Fontaine classification (stage IIa, IIb, III and IV). No significant differences were found between groups (Figure 4).

**Figure 5:**
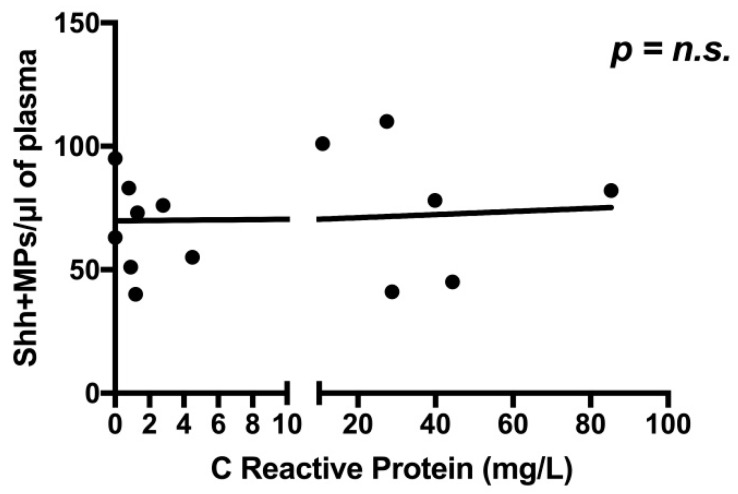
Global number of Shh + MPs according to C Reactive Protein (CRP) levels. Black horizontal line indicates the interpolate curve.

In subjects with PAD, inflammation might be an additional driver for the production of Shh + MPs. Indeed, it is established that circulating levels of MPs are increased in pathologies associated with systemic or local inflammation [15] and, since inflammation is characterized by interactions among platelets, leukocytes, and endothelial cells, Shh + MPs might be produced by these cell types in the setting of vascular diseases. For these reasons, we looked for a possible association between the number of Shh + MPs and the blood levels of C Reactive Protein (CRP), a prototypical inflammatory marker. However, no statistically significant association was found (this analysis was performed only on 14 PAD subjects, since CRP levels were available only for these patients) (Figure 5).

**Figure 6:**
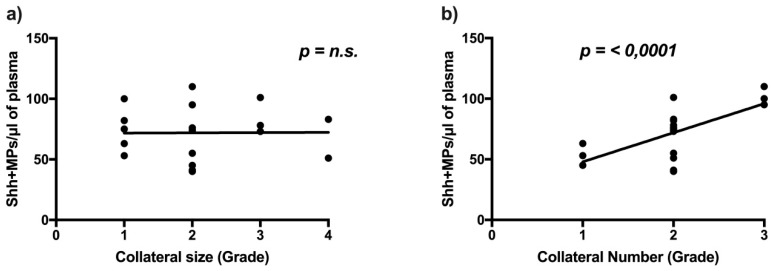
Global number of Shh + MPs in PAD patients according to size (**a**) and number (**b**) of collateral vessels, visualized by the mean of CT angiography. For collateral size, Grade 1 corresponds to ≤5 small collateral vessels, Grade 2 to >5 small collateral vessels, Grade 3 to ≤5 large ± small collateral vessels, and Grade 4 was defined to >5 large ± small collateral vessels. For collateral number, Category 1 corresponds to 0–3 collateral vessels, Category 2 to 4–7 collateral vessels, and Category 3 to ≥8 collateral vessels. Black horizontal lines in panels indicate the interpolate curve.

Since Shh is a potent prototypical angiogenic agent [3] we hypothesized that higher levels of circulating Shh + MPs may be associated with increased number of collateral vessels in the ischemic limb. To test this hypothesis, we analyzed the Computed Tomography (CT) angiography data of eighteen PAD patients and looked for a possible association between number of Shh + MPs and collateral vessel size and number (Figure 6). We found a statistically significant association between number of collaterals and number of Shh + MPs, with PAD subjects that had a number of circulating Shh + MPs around 100/μL exhibiting ≥8 collateral vessels, and PAD subjects that had Shh + MPs around 50/μL displaying only 0–3 collateral vessels. No association was bound between the number of Shh + MPs and collateral size.

**Figure 7:**
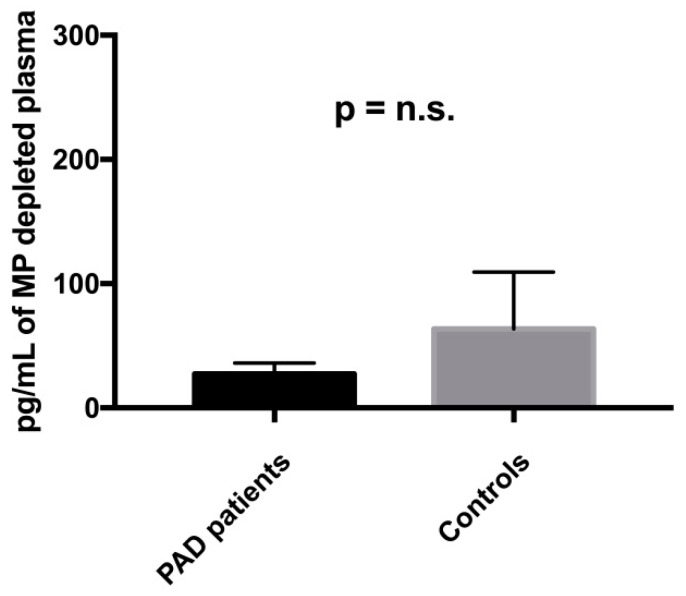
Levels of Shh protein in MP-depleted plasma of PAD patients and controls.

Finally, we used microparticle-depleted (MP-depleted) plasma to assess the levels of Shh protein in subjects with PAD and controls. We found detectable levels of Shh protein in the MP-depleted plasma of all investigated individuals. However, there was no difference between subjects with PAD and controls (47.5 ± 8.5 pg/mL vs. 53.5 ± 25.8 pg/mL, *p* = n.s.) (Figure 7).

## 3. Discussion

This study was aimed to evaluate whether upregulation of Shh occurs in humans in the setting of ischemia. We decided to study patients with PAD, because this is a prototypical ischemic pathology and is characterized by the fact that important muscular masses are exposed to severe degrees of ischemia over a prolonged period of time. We found that the number of MPs bearing Shh is significantly increased in subjects with PAD compared to controls. Importantly, we did not find an increment in the global number of circulating MPs of endothelial, platelet, leukocyte, or erythrocyte origin, but only in those MPs that carry Shh, thus suggesting a specific role for this type of MPs in this pathological condition. Importantly, the number of Shh + MPs in our PAD cohort is not affected by the possible association with CAD and/or stroke, which indicates that these MPs are increased in PAD subjects independently on other ischemic cardiovascular diseases.

We have not investigated the mechanisms responsible for the increased production of Shh + MPs in subjects with PAD. However, it is likely that hypoxia and ischemia are the main drivers of activation of the Shh pathway in these patients. Indeed, we have previously demonstrated, in a number of experimental animal models, that ischemia has the ability to reactivate the Hedgehog (Hh) signaling pathway. For instance, upon induction of hindlimb ischemia in mice, there is strong upregulation of Shh within the ischemic area [12]. Recapitulation of the Shh pathway also occurs upon induction of myocardial ischemia, as well as in experimental models of diabetic vascular disease, in rats and rabbits [13,14]. Nonetheless, how ischemia leads to Hh activation is still not completely understood. Experimental data have shown that hypoxia per se—independently on ischemia—is able to induce a rapid systemic Hh response in various organs of adult mice, with the Hh response being preceded by the accumulation of the master transcriptional regulator of hypoxia Hypoxia Inducible Factor-1α (HIF-1α) [16]. Pharmacological inhibition, knockdown, or genetic ablation of HIF-1α abolishes Hh activation in hypoxic tissues [16]. Regarding the role of inflammation on the production of Shh + MPs in the setting of PAD, it should be said that this may certainly be a possible mechanism, based on the notion that pathologies associated with systemic or local inflammation are commonly associated with MP production [15] and that, in PAD, the production of Shh + MPs might be finalized to limit inflammation [17]. However, in our cohort of PAD patients, we have been unable to detect an association between the number of circulating Shh + MPs and the blood levels of the prototypical inflammatory marker CRP.

In ischemic conditions, the reactivation of the Shh pathway is functionally important, since Shh is a potent angiogenic agent able to upregulate various families of angiogenic growth factors and is considered an important pathway for tissue repair and regeneration [3,18,19,20]. Previous experimental studies have shown that Shh carried by MPs has the ability to induce NO release from ECs, trigger changes in the expression and phosphorylation of enzymes related to the NO pathway, and decrease production of reactive oxygen species [1]. It has also been reported that Shh + MPs are capable to induce the formation of capillary-like structures in vitro and increase the expression of proangiogenic factors in cultured ECs [21]. More recently, it has been shown that Shh + MPs are able to modulate neovascularization in a murine model of hind-limb ischemia, correct angiotensin II-induced hypertension and endothelial dysfunction in mice, and reduce infarct size in a rat model of cardiac ischemia-reperfusion injury [10,22,23]. Interestingly, we have found that the number of Shh + MPs correlates in a statistically significant manner with the number of collateral vessels, assessed by CT angiography in a subset of our PAD cohort. In this scenario, our findings are consistent with the hypothesis that Shh + MPs might be an underestimated player in the pathophysiology of ischemic disease and support the concept that MPs harboring Shh might contribute to the regulation of angiogenesis in the ischemic limb. On the other hand, we have not found an association between Shh + MPs and disease stage. However, disease stage does not exclusively depend on the number of collateral vessels detected by CT-angiography, but is instead the results of a number of demographical, clinical, and pathobiological factors. Certainly, the precise role of Shh + MPs and their clinical significance in PAD patients remain to be elucidated. 

We also determined the concentration of Shh protein in MP-depleted plasma of subjects with PAD and controls, finding no differences between the two groups. This is an interesting finding which suggests that, in the setting of ischemia, the call for Shh recapitulation does not lead to the secretion of protein into the blood, where it would be inactivated by inhibitory proteins, but to the binding of the protein to the membrane of MPs, in order to warrant its activity and the delivery of the message in a protected environment. This would be consistent with previous reports in the literature, that have clearly demonstrated that Shh carried by MPs is functionally active and has its main target in ECs. In particular, using in vitro assays, it has been shown that Shh + MPs are able to stimulate ECs to release NO, produce proangiogenic factors, and form capillary-like structures [21]. Shh + MPs have shown their functional potentials also in vivo, when injected into rodents to stimulate angiogenesis [10], correct endothelial dysfunction [22], and reduce myocardial infarct size [23]. Thus, anchoring Shh on the surface of MPs might be a strategy used by producing cells to protect Shh from plasmatic inhibitory proteins, provide the lipid and protein environment necessary to maintain Shh activity, and deliver the active protein to target cells.

This study has some potential limitations. First, it has been conducted on a relatively number of patients and thus deserves confirmation in larger samples. Also, it will be important to confirm these findings using an additional technique, i.e., Western blotting. We have not assessed the functional properties of Shh + MPs. In particular, it would be interesting to use in vitro and in vivo models of angiogenesis to determine whether MPs isolated from the plasma of subjects with PAD have different angiogenic capacities compared to MPs isolated from the plasma of subjects without PAD. Finally, it should be mentioned that previous data by other groups have found differences between PAD patients and controls in terms of number of circulating MPs of endothelial [24] or platelet [25] origin, in contrast with our findings. However, while in such previous studies control patients were subjects without cardiovascular diseases, or even healthy volunteers, our controls were subjects with carotid plaques. 

In conclusion, this study shows that Shh bound to MPs is increased in subjects with PAD, while plasmatic Shh is not. Our findings provide novel information on Shh signaling in the course of ischemia in humans, with potentially important fundamental and clinical implications.

## 4. Materials and Methods

### 4.1. Patients 

PAD subjects (*n* = 50) and controls (*n* = 50) were enrolled among patients consecutively admitted to the Vascular Medicine Outpatient Clinic of the A. Gemelli University Hospital of Rome, Italy and the Angiology Unit of the San Giacomo Hospital of Castelfranco Veneto, Italy, between 1 August 2015 and 30 September 2016. Inclusion criteria for patients were age >18 years, Caucasian race, and presence of clinically relevant PAD. Diagnosis of PAD was made in accordance with the criteria established by the Ad Hoc Committee on Reporting Standards of the Society for Vascular Surgery and the International Society for Cardiovascular Surgery [26]. Severity of the disease was determined using the Leriche–Fontaine classification (stage IIa, IIb, III and IV). At the time of inclusion, each subject underwent clinical examination, ankle-brachial index (ABI) measurement and lower limb Doppler ultrasound. Inclusion criteria for controls were age >18 years, Caucasian race and no clinical and ultrasound evidence of PAD. Controls were mainly identified among individuals undergoing clinical and ultrasonographic follow-up for asymptomatic carotid plaque at the above-mentioned vascular centers. In all controls, PAD was excluded by appropriate clinical interview, physical examination, ankle-brachial index (ABI) measurement, and lower limb Doppler ultrasound. For both groups, exclusion criteria were cancer (current or previous), chronic inflammatory diseases, infectious diseases, autoimmune diseases, thrombotic diseases of the venous system, and thrombophilia. The study was conducted in accordance with the ethical standards of the Helsinki declaration and its later amendments and with the ethical standards and approval of the Università Cattolica del Sacro Cuore/Fondazione Policlinico Universitario A. Gemelli IRCCS (protocol number 1827/15; 23 March 2015). Informed consent was obtained from all patients included in the study.

### 4.2. Analysis of Collateral Vessels 

Multidetector computed tomography (CT) angiography was used to assess size and number of collateral vessels in a subset of PAD patients (those with fully available images, *n* = 18), by the means of a validating scoring system, based on previous studies [27]. One radiologist (Roberto Iezzi), blinded to the characteristics of the patients, reviewed the images. The size of the collateral vessels was categorized as large if they occupied >25% of the length of the imaged thigh and >50% of the diameter of the superficial femoral artery (SFA). For the collateral vessel size, grade 1 was defined as ≤5 small collateral vessels, grade 2 was defined as >5 small collateral vessels, grade 3 was defined as ≤5 large ± small collateral vessels, and grade 4 was defined as >5 large ± small collateral vessels. For the number of collateral vessels, category 1 was defined as 0–3 collaterals, category 2 was defined as 4–7 collaterals, and category 3 was defined as ≥8 collaterals.

### 4.3. MP Analysis 

**Figure 8:**
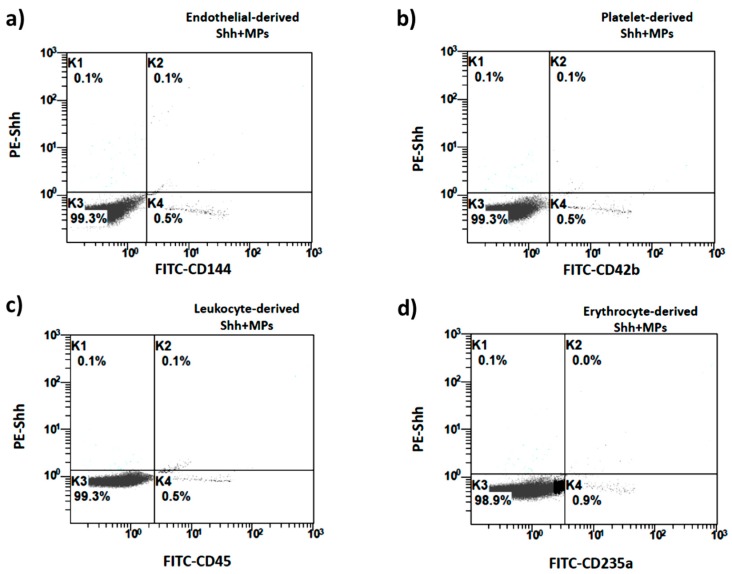
Size-selected events are plotted according to their fluorescence for specific CD144, CD42b, CD45, CD235a, and Shh binding. Events included in the K4 section were considered Shh + EMPs (**a**), Shh + PMPs (**b**), Shh + LMPs (**c**), and Shh + ErMPs (**d**).

MPs isolation and analysis was performed accordingly with current methodological guidelines [28]. Blood was collected into sodium citrate vacutainer tubes from a peripheral vein using a 21-gauge needle and processed within 1 h from the from the withdrawal. Samples were centrifuged at 450× *g* for 20 min at room temperature to collect platelet-rich plasma (PRP) and then at 1500× *g* for 20 min to generate platelet-free plasma (PFP). Analysis were conducted using a FC500 Flow Cytometer (Beckman Coulter, Brea, CA, USA) equipped with 2 laser lines (488 and 633 nm). The cytometer was preliminary calibrated by using the fluorescent Megamix beads (Biocytex, Marseille, France) covering the MP (0.5 and 0.9 µm) and platelet size ranges (0.9 and 3 µm). The upper and the outer limits of MP gate were established just above the size of the 0.9 µm beads in a forward (FS) and side scatter (SS), the lower limit was the noise threshold of the instrument, and an absolute minimum threshold of 1 was set for the SS in order to reduce the background noise. MPs were identified as particles between 0.1 and 1 µm in size according to their light scattering. A total of 150,000 events were acquired for each sample. Selected samples were serially diluted in order to avoid coincidence and reduce artifacts. EMPs, PMPs, LMPs, and ErMPs were defined as vesicles positive for either CD144, CD42b, CD45, or CD235, respectively. Shh + MPs were identified as vesicles positive for Shh. Shh + EMPs were defined as vesicles double positive for CD144 and Shh (Figure 8a). Shh + PMPs were defined as vesicles double positive for CD42b and Shh (Figure 8b). Shh + LMPs were defined as vesicles double positive for CD45 and Shh (Figure 8c). Shh + ErMPs were defined as vesicles double positive for CD235a and Shh (Figure 8d). For Shh labelling, 150 µL of PFP were incubated for 30 min in the dark at room temperature with 3 µL (1:50) of PE-labeled anti-Shh antiboby (R&D Systems, Minneapolis, MN, USA). For CD144 labelling, 3 µL (1:50) of FITC-labeled anti-CD144 antibody (BD Pharmigen, Franklin Lakes, NJ, USA) were used. For CD42b labelling, 3 µL of FITC-labeled anti-CD42b antibody (Beckman Coulter, Brea, CA, USA) were used. For CD45 labelling, 3 µL of FITC-labeled anti-CD45 antibody (Beckman Coulter, Brea, CA, USA) were used. For CD235a labelling, 3 µL of FITC-labeled anti-CD235 antibody (Beckman Coulter, Brea, CA, USA) were used. Isotope controls were used in all experiments. An equal volume of Flow Count fluorospheres (Beckman Coulter, Brea, CA, USA) was added to the samples in order to determine MP concentration. Values are reported as number of MPs in 1µL of PFP (number/µL).

### 4.4. Plasma Collection, MP Depletion of Plasma, and Determination of Shh Protein Concentration 

Blood was collected from PAD patients and controls into sodium citrate vacutainer tubes. Plasma was obtained by centrifugation of blood at 1570× *g* at 4 °C for 20 min. To generate MP-depleted plasma, we used the method described by Crowford and coll. [24]. In brief, the top 90% of plasma was collected and first centrifuged again at 1570× *g* at 4 °C for 20 min and then depleted of MPs through further high-speed centrifugation. The supernatant was kept as MP-depleted plasma. Shh levels were determined by using a dedicated ELISA test (R&D Systems, Minneapolis, MN, USA). 

### 4.5. Statistical Analysis 

Results are presented as mean ± SD. Since data were not always normally distributed, the non-parametric Mann-Whitney U test was used for comparison of two data sets. Correlation between the number of circulating MPs and clinical variables was evaluated by Pearson’s correlation test. Differences were considered statistically significant for *p* < 0.05. Calculations were performed with Prism 7 (GraphPad Software; https://www.graphpad.com/scientific-software/prism/) and IBM SPSS Statistics for windows, Version 20.0, released 2011 (IBM Corp; https://www.ibm.com/analytics/it/it/technology/spss/).

## Figures and Tables

**Table 1:** Demographic and clinical characteristics of the studied population.

Variables		PAD	Controls	*p*
Years of age (mean ± SD)		71.4 ± 9.4	70.3 ± 8.2	n.s.
Males, n (%)		37 (74.0)	27 (54.0)	<0.05
Smokers, n (%)		17 (34.0)	7 (14.0)	<0.01
Diabetes, n (%)		22 (44.0)	17 (34.0)	n.s.
Dyslipidemia, n (%)		36 (72.0)	30 (60.0)	n.s.
Hypertension, n (%)		40 (80.0)	40 (80.0)	n.s.
Previous MI, n (%)		21 (42.0)	4 (8.0)	<0.01
Previous Stroke, n (%)		6 (12.0)	0 (0.0)	<0.01
Therapy	SAPT, n (%)	33 (66.0)	24 (48.0)	0.04
	DAPT, n (%)	9 (18.0)	0 (0.0)	<0.01
	Statins, n (%)	36 (72.0)	28 (56.0)	n.s.
	ACE inhibitors/ARB, n (%)	27 (54.0)	26 (52.0)	n.s.
	Beta-blockers, n (%)	23 (46.0)	19 (38.0)	n.s.

n: number; MI: myocardial infarction; SAPT: single antiplatelet therapy; DAPT: dual antiplatelet therapy; ACE: angiotensin converting enzyme; ARB: angiotensin II receptor blockers.

**Table 2:** Number and type of circulating MPs in PAD patients and controls.

Type of MPs	PAD	Controls	*p*
Shh + MPs/μL (mean ± SD)	70.5 ± 19.0	27.2 ± 8.5	*p* < 0.001
EMPs/μL (mean ± SD)	138.2 ± 49.2	130.6 ± 62.2	n.s
Shh + EMPs/μL (mean ± SD)	22.3 ± 21.8	9.2 ± 6.0	*p* < 0.001
PMPs/μL (mean ± SD)	143.9 ± 77.1	126.1 ± 61.6	*n.s*
Shh + PMPs/μL (mean ± SD)	17.3 ± 10.1	9.9 ± 4.6	*p* < 0.001
LMPs/μL (mean ± SD)	126.9 ± 116.6	130.5 ± 79.0	0.09
Shh + LMPs/μL (mean ± SD)	15.2 ± 7.9	9.0 ± 3.9	*p* < 0.001
ErMPs/µL (mean ± SD)	315.5 ± 238.7	271.6 ± 159.5	*n.s.*
Shh + ErMPs/µL (mean ± SD)	20.0 ± 11.8	9.8 ± 4.6	*p* < 0.001
